# Integrated systems improve soil microclimate, soybean photosynthesis and growth

**DOI:** 10.3389/fpls.2024.1484315

**Published:** 2025-01-09

**Authors:** Luciana Maria da Silva, Eduardo Habermann, Kátia Aparecida de Pinho Costa, Adriano Carvalho Costa, João Antônio Gonçalves e Silva, Eduardo da Costa Severiano, Lourival Vilela, Fabiano Guimarães Silva, Alessandro Guerra da Silva, Bruno de Souza Marques, Fabrício Rodrigues, Carlos Alberto Martinez

**Affiliations:** ^1^ Department of Agricultural Sciences/Agronomy, Goiano Federal Institute (IF Goiano), Rio Verde, Brazil; ^2^ Department of Biology, School of Philosophy, Science and Literature (FFCLRP), University of São Paulo, Ribeirão Preto, Brazil; ^3^ Embrapa Cerrados, Planaltina, Brazil; ^4^ Department of Agronomy, University of Rio Verde, Rio Verde, Brazil; ^5^ Department of Plant Production, Universidade Estadual de Goiás, Ipameri, Brazil

**Keywords:** climate change, legumes, plant physiology, sustainability, triple intercropping

## Abstract

This study aimed to compare the conventional soybean (*Glycine max* L.) cultivation method with integrated systems in an Latossolo Vermelho Acriférrico típico and how these systems affect soil cover biomass production, initial nutrient concentration in plant residues, soil respiration and microclimate, as well as soybean growth, physiology and productivity. A comparative analysis of microclimate and soil respiration, plant physiology, and growth was conducted between a conventional soybean monoculture (soybean grown without plant residues on the soil from the previous crop) and soybean grown in soil containing maize residues. Additionally, experiments were conducted to evaluate the effect of monocultures and previous integration between maize, three cultivars of *Panicum maximum* (Zuri, Tamani, and Quênia guinea grass) and Pigeon pea (*Cajanus cajan* cv. BRS Mandarim) on soil health, physiological aspects, and soybean production. Our results indicated that all cultivars of *Panicum maximum* can be used in integrated systems. The triple consortium resulted in greater production of ground cover biomass and a higher concentration of nitrogen, phosphorus, potassium and sulphur, which contributed to lower soil temperature and greater humidity, without a concomitant increase in soil respiration. Consequently, soybeans grown in the resulting integrated systems cover biomass showed a higher net photosynthesis rate and increased leaf chlorophyll index, resulting in taller plants, with higher above-ground biomass production and 21.0% and a 36.8% increase in grain yield when compared to soybean cultivated on maize biomass and on soil without cover residue, respectively. The data presented in this study demonstrated that integrated systems, with the presence of grasses and legumes, improve soil climatic conditions and nutrient availability, enhancing soybean physiology and productivity characteristics, thus contributing to the sustainability of agricultural production, even in the short term. Further long-term research is strongly recommended.

## 1 Introduction

Anthropogenic climate change has triggered significant modifications in Earth’s climate patterns by modifying temperature and precipitation patterns (Bhatti et al., 2024). With predictions indicating a worsening trend in the next years (IPCC, 2019). All these alterations in climate have profound impacts on agriculture and livestock, particularly impacting countries heavily dependent on agriculture for economic gains (Guo et al., 2022). Therefore, implementing strategies to mitigate climate change and foster resilient cultivation practices becomes paramount for safeguarding future food security.

The agricultural sector constitutes a substantial source of greenhouse gas (GHG) emissions. In this sector, GHG mainly originates from changes in land use, ruminant metabolism, and nitrogen fertilization (Ma et al., 2024). In the broader framework of global initiatives aimed at mitigating greenhouse gas emissions, the adoption of cultivation practices designed to mitigate these emissions assumes critical importance. Within this spectrum of mitigation strategies, an integrated crop-livestock system emerges as a particularly promising agricultural approach (Delandmeter et al., 2024). These systems possess the potential to not only reduce fertilizer consumption but also improve the carbon sink capacity of crops, increase plant resilience against abiotic stresses (Han et al., 2024; Silveira et al., 2024), and improve productivity and soil fertility by promoting greater nutrient cycling, when compared to monoculture systems (Farias et al., 2020; Muniz et al., 2021; Vendramini et al., 2023; Silva et al., 2024b).

Integrated crop-livestock systems involve the integrated execution of agricultural and livestock activities, either in rotation, succession, or consortium, within the same area but at different times (Nunes et al., 2021). In the last decades, attention has been directed toward assessing the impact of integrated systems on soil characteristics and productivity (Lima et al., 2023). These systems have proven effective in pasture recovery (Santos et al., 2023; Damian et al., 2023) and improvements in soil health (Silva et al., 2024b). Soil health is the soil’s capacity to function as a vital ecosystem that supports biological productivity, preserves environmental quality, and enhances the well-being of plants and animals (Barbosa et al., 2023; Serafim et al., 2023; Carvalho et al., 2024). Consequently, with improvements in soil health, the productivity of crops grown in succession, such as soybean, has shown significant increases. Brito et al. (2023) observed a 21% increase in soybean grain yield when grown in an integrated system, after maize was intercropped with forage, when compared to growing after maize in monoculture. Silva et al. (2024c), on the other hand, found that integrated systems improve soil fertility, which increased the cumulative grain yield of soybeans by 15%.

Although the benefits of integrated systems on productivity have been well-established in the scientific literature, there remains a notable gap in understanding the physiological advantages experienced by plants in such cultivation systems and few physiological mechanisms responsible for enhancing plant growth under integrated systems are known. Evidence indicates that plants may experience higher soil moisture levels during the growing season and drought periods because crop residues cover the soil (Silva et al., 2024a) and protect the soil from solar radiation. Therefore, plants growing under these conditions can maintain an elevated stomatal conductance, transpiration flux, nutrient absorption, and photosynthesis. Moreover, the additional availability of nutrients in the soil resulting from the decay of plant residues may result in higher absorption of these nutrients, presumably increasing chlorophyll biosynthesis, photosynthesis, and biomass accumulation. Due to the higher photosynthesis and decay of crop residues, soil carbon stocks increase under crop-livestock systems, increasing overall soil metabolism (Carvalho et al., 2024; Silva et al., 2024b). Improvements in soil microbial conditions are also possible, even in the short term, in no-till systems, when compared to conventional cultivation (Mirzavand et al., 2022).

Many different strategies can be used to conduct a successfully integrated system. While the production of plant residues resulting from production from double cropping (e.g., maize + forage) for soybean no-till system is well established (Costa et al., 2020; Dias et al., 2020; Santos et al., 2021; Coelho et al., 2023; Silva et al., 2024c), there is a lack of information regarding the production of plant residues from triple cropping (e.g., maize + forage + legume), particularly when utilizing legumes in the system. Therefore, the ecosystem services for the soil-plant system, such as plant nutrition (Jensen et al., 2020; Gut et al., 2022), soil conservation (Kebede, 2021; Silva et al., 2022; Witcombe et al., 2023), soil microbial (Mirzavand et al., 2022), soil health (Yan et al., 2023), and maximization of productivity, quality, and profitability (Pereira et al., 2023; Souza et al., 2023) resulting from an integrated triple cropping system remains scarce in the literature.

Our study aimed to compare the conventional soybean cultivation method (soybean grown without plant residues on the soil from the previous crop) with alternative regimes in which soybeans are grown over soil residues from monoculture or a prior triple consortium of maize, three cultivars of *Panicum maximum* (Zuri, Tamani, and Quênia guinea grass), and Pigeon pea (*Cajanus cajan* cv. BRS Mandarim), and how these systems affect soil cover biomass production, initial nutrient concentration in plant residues, soil respiration and microclimate, as well as soybean growth, physiology and productivity. We hypothesize that soil cover crop residues would have a higher nutrient concentration. Growing soybean on these plant residues would result in increased photosynthetic rates, chlorophyll index, aboveground biomass production, soil respiration, and grain yield compared to conventional soybean cultivation method. Furthermore, residues from a triple intercropping system would lead to improved soil microclimatic conditions, higher photosynthetic rates of soybean, and increased aboveground biomass productivity, as well as a higher soybean grain yield, compared to monoculture residues.

## 2 Materials and methods

### 2.1 Description of the area

**Figure 1:**
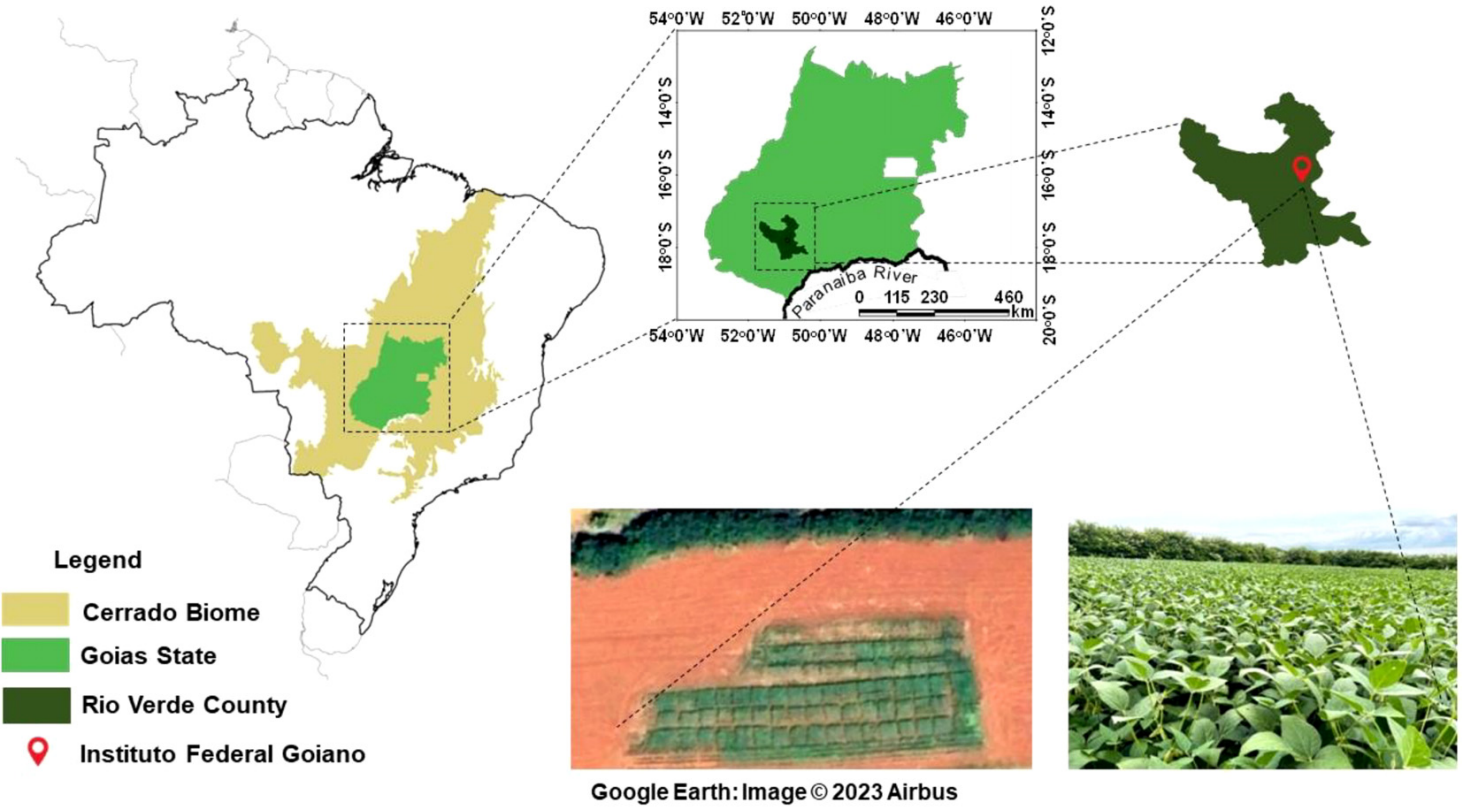
Experimental area during the experiment in Rio Verde, Goiás, Brazil.

The experiment was conducted at the Goiano Federal Institute (IF Goiano), Goiás, Brazil, under the coordinates 17° 48’ 53’’ S and 50° 54’ 02’’ W, at an altitude of 748m (**Figure 1**), from January 2021 to March 2022, covering two cropping seasons. In the first crop season, maize and tropical forages were cultivated in monoculture and intercropping systems for silage production, and in the second crop season, soybean were grown on the residues from monocultures and intercropping systems. The experimental area has a history of over ten years of integrated crop-livestock system management.

Before the experiment was set up, soil samples were collected from the 0-20 cm layer for physical-chemical characterization of the soil. The soil in the experimental area was characterized as Latossolo Vermelho Acriférrico típico (Santos et al., 2018), Oxisol in the USA Keys of Soil Taxonomy (Soil Survey Staff, 2014), with 562 g kg^-1^ of clay; 94 g kg^-1^ of silt and 344 g kg^-1^ sand; pH in CaCl_2_: 5.5; calcium (Ca): 2.70 cmol_c_ dm^-3^; magnesium (Mg): 1.40 cmol_c_ dm^-3^; aluminium (Al): 0.01 cmol_c_ dm^-3^; hydrogen (H) + Al: 3.41 cmol_c_ dm^-3^; potassium (K): 0.70 cmol_c_ dm^-3^; cation exchange capacity: 8.21 cmol_c_ dm^-3^; current base saturation of the soil (V1): 58.5%; phosphorus (P) (mehlich): 3.5 mg dm^-3^; sulfur (S): 8.6 mg dm^-3^; copper (Cu): 3.5 mg dm^-3^; zinc (Zn): 1.0 mg dm^-3^; iron (Fe): 17.2 mg dm^-3^; organic matter (O.M.): 34.7 g dm^-3^.

**Figure 2:**
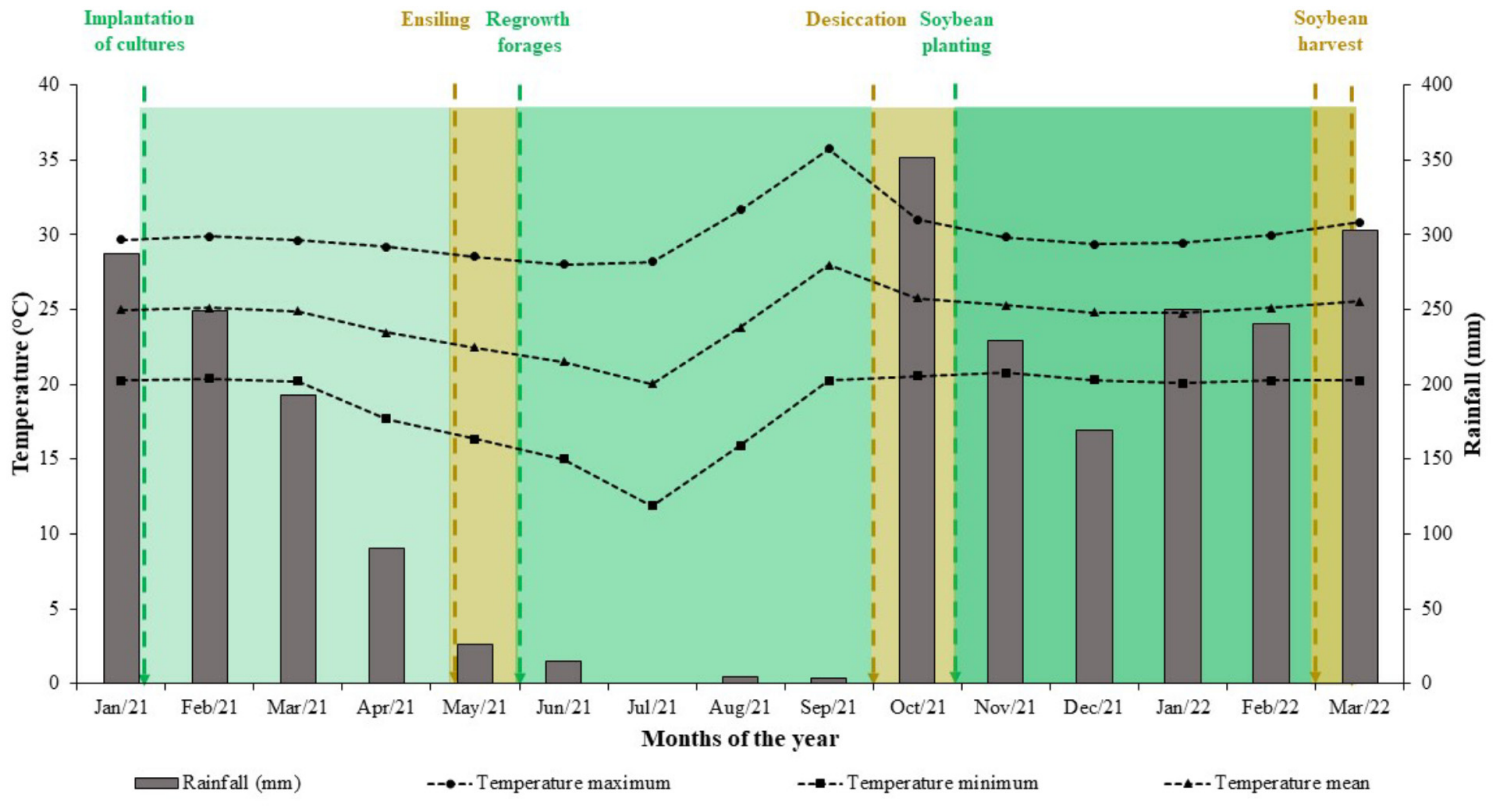
Rainfall and monthly temperatures recorded from January 2021 to March 2022 in Rio Verde, Goiás State, Brazil.The bars show the accumulated precipitation for each month, while the symbols + lines represent the average monthly temperature, maximum temperature, and minimum temperature.

During the experimental period, were monitored the precipitation amount and the maximum, average, and minimum monthly temperatures (**Figure 2**). Were observed an average temperature of 24.3°C and total annual precipitation of 2410 mm, with an regular distribution of rainfall.

Initially, on January 28, 2021, were conducted monoculture and triple intercropping of maize (*Zea mays* L. hybrid P4285), forage grasses of the genus *Panicum* (*Panicum maximum* cv. BRS Zuri guinea grass, BRS Tamani guinea grass, and BRS Quênia guinea grass) and the legume Pigeon pea (*Cajanus cajan* cv. BRS Mandarim) to produce silage in integrated systems.

At planting, 150 kg ha^-1^ of P_2_O_5_ in the form of single superphosphate and 20 kg ha^-1^ of FTE BR 12 (9% zinc (Zn), 1.8% boron (B), 0.8% copper (Cu), 2% manganese (Mn), 3.5% iron (Fe), and 0.1% molybdenum (Mo)) from Frits sources were applied in the planting furrow. Topdressing fertilization was carried out when the maize plants reached the stages of three and six fully developed leaves, with two topdressings applied to the soil, totaling 150 kg ha^-1^ of N and 80 kg ha^-1^ of K_2_O, from urea and potassium chloride sources, respectively. The same quantities of urea and potassium chloride were applied to the monocultures of grasses. For the intercropped systems, only half the urea dose was applied (75 kg ha^-1^ of N), aiming for the utilization of biological nitrogen fixation by the Pigeon pea, along with 80 kg ha^-1^ of K_2_O. For Pigeon pea in monoculture, only potassium fertilization was used with an application of 80 kg ha^-1^ of K_2_O.

After harvesting the monocultures and the intercropping systems (triple intercropping) for silage production, the forage regrowth occurred, generating plant residues for soil cover, which were used for no-till planting of soybean.

**Figure 3:**
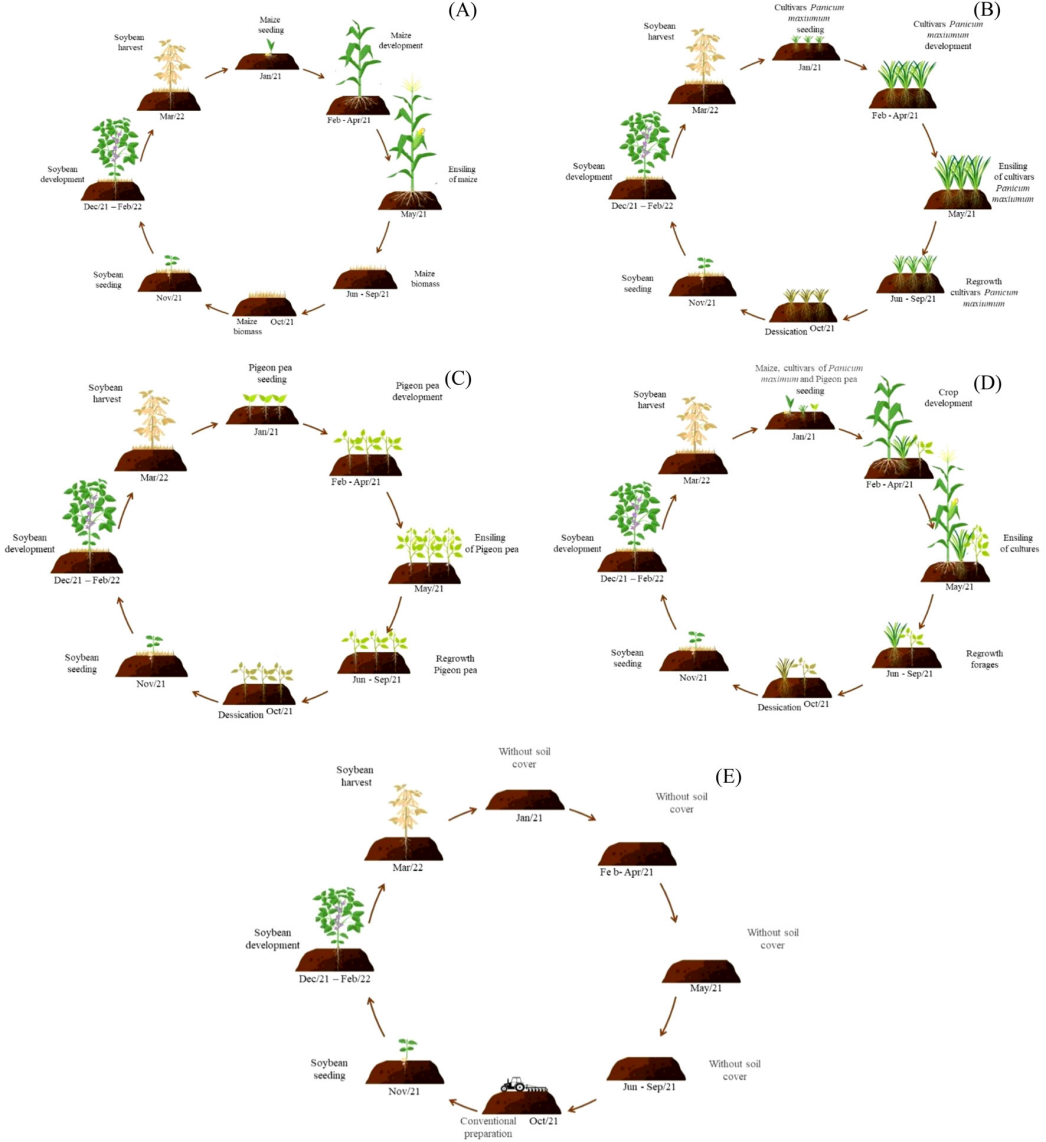
Scheme of cultivation systems: maize in monoculture **(A)**, cultivars *Panicum maximum* (Zuri, Tamani and Quênia guinea grass) in monoculture **(B)**, Pigeon pea in monoculture **(C)**, maize intercropped with cultivars Panicum maximum and Pigeon pea **(D)**, and conventional soybean cultivation without plant residues covering the soil **(E)**.

In this way, soybean plants were cultivated under the following combinations of plant residues: soybean cultivated on sole crop maize residues, soybean cultivated on sole crop Zuri guinea grass residues, soybean cultivated on sole crop Tamani guinea grass residues, soybean cultivated on sole crop Quênia guinea grass residues, soybean cultivated on sole crop Pigeon pea residues, soybean cultivated on intercropping residues from maize, Zuri guinea grass and Pigeon pea, soybean cultivated on intercropping residues from maize, Tamani guinea grass and Pigeon pea, soybean cultivated on intercropping residues from maize, Quênia guinea grass and Pigeon pea. Soybean was also sown in conventional cultivation method (without plant residues over the soil) (**Figure 3**). The experiment was organized in a randomized complete block design, with three replications, totaling nine treatments.

Were conducted the sowing of all treatments manually on the same day (January 28, 2021). For monocultures, crops were sown in five rows 4 m long, with a spacing of 0.90 m between rows. In the intercrops, maize was sown in five rows spaced 0.90 m apart, with forage crops of the genus *Panicum* (Zuri guinea grass, Tamani guinea grass, and Quênia guinea grass) and legume (Pigeon pea) sown between rows 0.30 m from the row of maize, 2 cm deep. In all treatments, maize seeds were sown 3 cm deep.

The harvest of maize and forages for silage production was conducted on May 10, 2021. At harvest, the crops were cut 20 cm above the ground, using a brush cutter. Dry mass production per hectare was 14,629 kg ha^-1^ (maize monoculture), 5,548 kg ha^-1^ (Zuri guinea grass monoculture), 4,211 kg ha^-1^ (Tamani guinea grass monoculture), 4,853 kg ha^-1^ (Quênia guinea grass monoculture), 24,219 kg ha^-1^ (maize intercropped with Zuri guinea grass and Pigeon pea), 22,882 kg ha^-1^ (maize intercropped with Tamani guinea grass and Pigeon pea) and 23,524 kg ha^-1^ (maize intercropped with Quênia guinea grass and Pigeon pea).

Subsequently, cultivars of the genus *Panicum* and Pigeon pea plants were cultivated during the off-season (between June and August), with successive cuts, simulating grazing. After the development of forage crops, desiccation was carried out in October 2021, aiming for the formation of plant residues for soybean planting. We used 3 L ha^-1^ of glyphosate herbicide for all forages in the desiccation process.

### 2.2 Soybean sowing

Before soybean sowing, ten soil samples were collected homogeneously within the plots (depth of 0 to 20 cm), and mixed to form a composite sample, and used to determine the chemical properties of the soil and perform fertilization recommendations (**Table 1**).

**Table 1:** Chemical characteristics of a Latossolo Vermelho Acriférrico típico at a depth of 0-20 cm before soybean sowing.

pH	Ca	Mg	Al	H+Al	K	CEC	P	Zn	Fe	Cu	V_1_	OM
CaCl_2_	—————— cmol_c_ dm^-3^ —————–						——— mg dm^-3^———				%	g kg^-1^
5.4	2.69	1.43	0.01	4.79	0.69	9.62	3.8	1.0	17.3	3.7	50.2	39.8

CEC, cation exchange capacity; P, Mehlich; V_1_, base saturation; OM, organic matter.

According to the recommendations obtained from the chemical analysis, one ton ha^-1^ of limestone filler (100% of the relative total neutralizing power) was applied throughout the experimental field, without incorporating limestone. Soybean (variety Bônus IPRO 8579) were sown in October 29, 2021 in a mechanized way, with row spacing of 0.50 m, with an average population of 240,000 plants ha^-1^ in the different cultivation systems. When sowing soybean, 180 kg ha^-1^ of P_2_O_5_ (simple superphosphate) was used in all treatments and applied in the planting furrow. Potassium was not applied in treatments containing plant residues to take advantage of nutrient cycling. In the soybean treatment grown without plant residues over soil, 85 kg ha^-1^ of K_2_O was applied to the potassium chloride source. Fungicide application was carried out 45 and 60 days after sowing (DAS) (dose of 0.4 L ha^-1^ of *Pyraclostrobin*). The soybean harvest was carried out on March 1, 2022.

### 2.3 Soil cover crop biomass analyses

#### 2.3.1 Biomass production

To determine the production of soil residues in each integrated system, we employed the quadrat method (0.50 × 0.50 m, 0.25 m^2^). Measurements were taken 28 days after the desiccation of maize, forages, and legumes. One quadrat was positioned on the soil in each plot, and litter was collected from each area. The plant material was then placed in a forced air circulation oven at 55°C until a constant weight was reached.

#### 2.3.2 Initial nutrient concentration

After drying the plant residues, the plant material was grounded in a knife mill with a 1 mm sieve and stored in plastic containers to determine the concentration of nutrients: nitrogen (N), phosphorus (P), potassium (K), calcium (Ca), magnesium (Mg) and sulfur (S) according to the methodology proposed by Malavolta et al. (1997).

### 2.4 Soil analyses

#### 2.4.1 Soil respiration

To investigate the influence of integrated management on soil respiration, we utilized a Li-8100A (LI-COR, NE, USA) featuring a manually operated chamber with a 20 cm diameter. The measurements were carried out 90 days after soybean sowing, within the time frame of 8 am to 11 am, following the protocol outlined by Gonzalez-Meler et al. (2017). In each plot, a polyvinyl chloride (PVC) collar measuring 20 cm in diameter was positioned in the central region between the soybean rows devoid of plants, for coupling soil CO_2_ Flux System. Each collar was inserted 5 cm below the ground level and protruded 5 cm above the ground surface.

#### 2.4.2 Soil microclimate

To comprehend the impacts of an integrated crop-livestock system on soil microclimate, we gathered five soil samples to assess soil moisture during the morning. Soil temperature was also evaluated at the time of sample collection. These samples were obtained 90 days after soybean sowing, coinciding with soil respiration measurements. Using a 20 cm deep soil probe, the samples were collected and sealed in plastic bags. Subsequently, the fresh weight (FW) of the samples was measured, followed by drying in an oven at 70 °C until a constant weight (dry weight, DW) was achieved.

Immediately after the soil respiration measurements, we collected one soil sample per plot near the polyvinyl chloride (PVC) collars. Soil moisture levels were computed based on the FW and DW values. The soil surface temperature was determined using an infrared surface thermometer (Testo 835–111/T1), while the soil temperature at a depth of 5 cm was measured with a soil thermometer. These soil sampling and temperature measurement procedures were carried out concurrently 90 days after soybean sowing and during the soil respiration assessments.

### 2.5 Plant analyses

#### 2.5.1 Leaf gas exchange

To investigate the impact of integrated management on the gas exchange of soybean plants, we employed a portable infrared gas analyzer, the LI-6800 (Licor, USA). Leaf gas exchange assessments were conducted on the central leaflet of an expanded leaf in the upper canopy region per plot. The measurements were carried out between 8 am and 11 am, 90 days after the soybean sowing. Throughout all measurements, we maintained constant conditions of radiation (2000 μmol m^−2^ s^−1^), CO_2_ concentration (400 ppm), and leaf temperature (25°C). The leaves were kept within the chamber until the parameters stabilized. We quantified the net photosynthesis rate, expressed in µmol m^−2^ s^−1^, stomatal conductance in mol m^−2^ s^−1^, and transpiration rate in mmol m^−2^ s^−1^.

#### 2.5.2 Leaf chlorophyll

The leaf chlorophyll index for soybean plants was determined using a CCM-200 Chlorophyll Meter (Opti-Science, Hudson, NH, USA) at midday and 90 days after sowing. Measurements were taken on ten expanded central leaflets (trifoliate leaves) on the adaxial surface per plot.

#### 2.5.3 Growth parameters and grain yield

At 104 days after sowing (DAS), we assessed the height (m) and aboveground biomass of the soybean plants. Plants were harvested from one linear meter in each plot, encompassing all aboveground plant material (stems, leaves, and pods). Plant material was subjected to drying in an oven until a stable dry weight was achieved, and we subsequently calculated the soybean aboveground dry mass, expressed kg ha^-1^. Grain yield was determined on March 1, 2022 (124 days after sowing), with the plants from the useful area of each plot being collected, and the values were expressed in kg ha^-1^.

### 2.6 Statistical analyses

The raw data underwent outlier analysis using GraphPad Prism 10 software. To adhere to the assumptions of analysis of variance (ANOVA), the data were examined for normality, homogeneity, and independence of residuals. Statistical analysis involved one-way ANOVA, followed by Tukey’s test (p<0.05). Correlation analysis was conducted to explore associations between parameters, and the results were visualized on a heatmap. The variables were standardized (X_i_− µ)/σ and subjected to principal component analysis (PCA). The R-3.1.1 software, incorporating packages such as “tidyverse” for database manipulation, “stats” for analysis, and “factoextra” for graphical representation, facilitated these analytical processes.

## 3 Results

**Figure 4:**
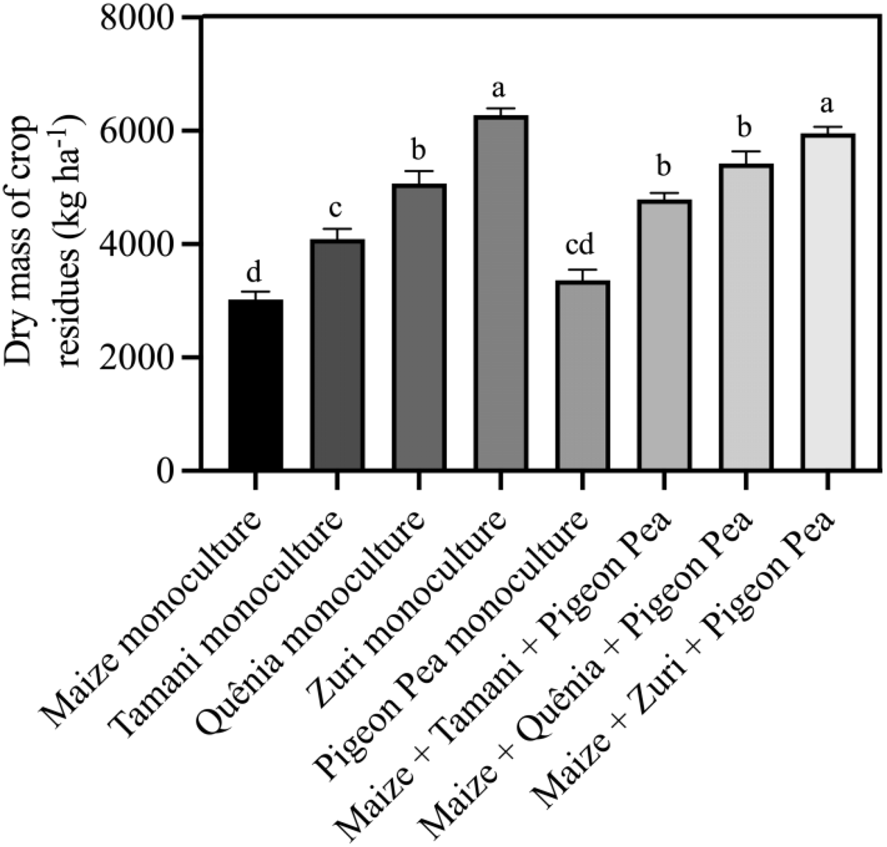
Dry mass of crop residues soil cover produced by different integration strategies. Different lowercase letters between bars indicate statistical difference (p<0.05) between treatments by ANOVA followed by the Tuckey test. Stacked bars above the columns indicated the standard error of the mean. Each bar is the average value of 3 replicates (n =3).

Our integration strategies resulted in different dry masses of plant residues for soil cover (**Figure 4**). Those integration strategies incorporating Zuri guinea grass were particularly effective in generating the highest of soil cover residues quantities. Interestingly, the biomass production remained similar whether Zuri guinea grass guinea grass was cultivated in monoculture or consortium with maize + Pigeon pea, with an increase of 50,6% in relation to the biomass of maize and increase 44,9% in relation to the soil cover biomass of Pigeon pea in monoculture, that presented the lowest soil cover biomass production among all integration strategies. Intermediate soil cover biomass productions were observed for treatments involving Tamani guinea grass guinea grass and Quênia guinea grass guinea grass, both in monoculture and when intercropped with maize and Pigeon pea.

**Figure 5:**
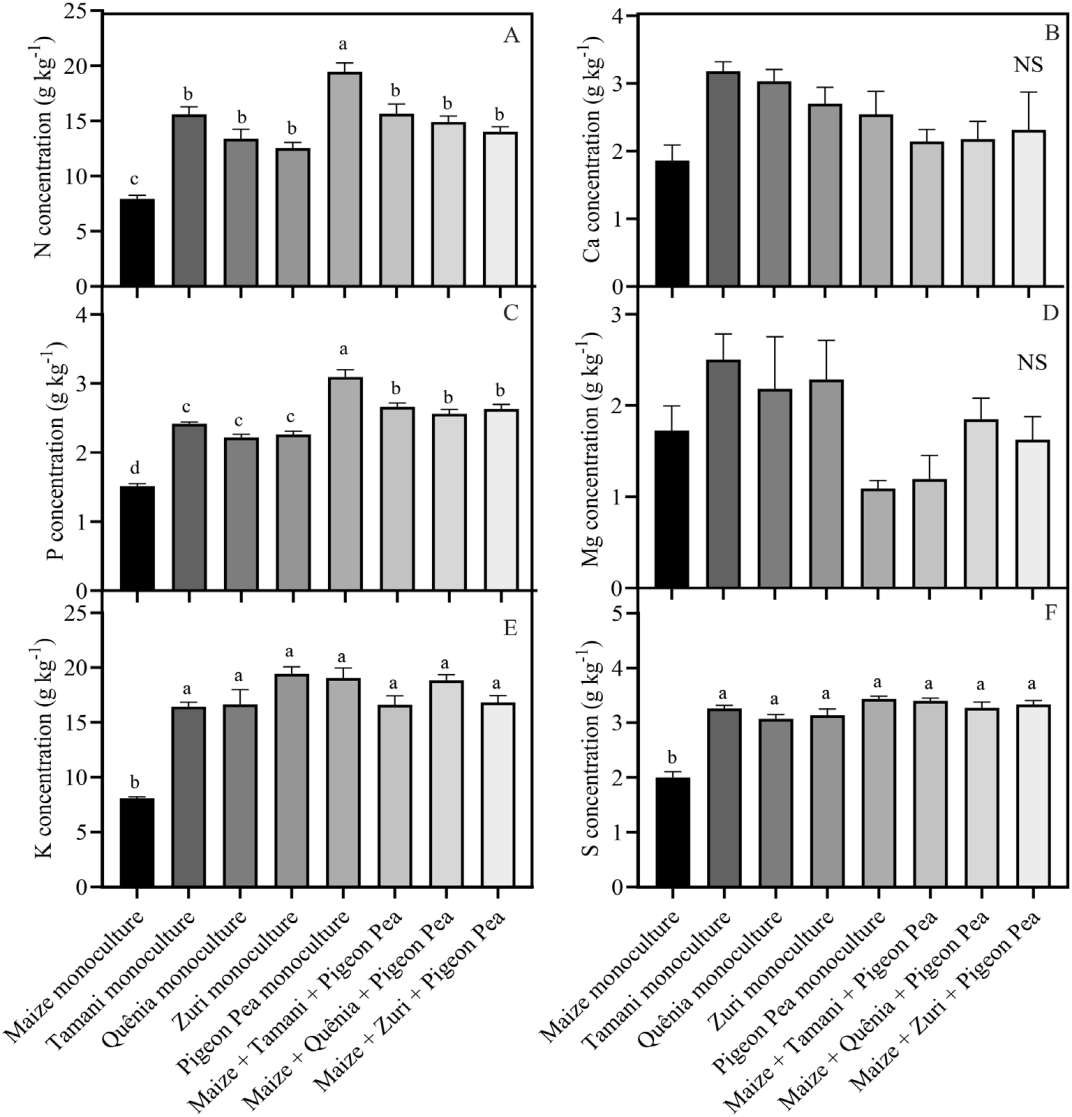
Nutrient concentration: N = nitrogen **(A)**, Ca = Calcium **(B)**, P = phosphorus **(C)**, Mg = magnesium **(D)**, K = potassium **(E)** and S = sulphur **(F)** of crop residues produced by different integration strategies. Different lowercase letters between bars indicate statistical difference (p<0.05) between treatments by ANOVA followed by the Tuckey test; NS in the top right corner indicates that there was no significant effect between the treatments by ANOVA followed by the Tuckey test. Stacked bars above the columns indicated the standard error of the mean. Each bar is the average value of 3 replicates (n =3).

The concentration of nutrients within the biomass generated by each integration strategy varied based on the composition of the biomass (**Figure 5**). Pigeon pea in monoculture showed the highest concentrations of N and P, with increases of 59.2% and 51.1%, respectively, compared to maize monoculture, which had the lowest concentrations. On the other hand, the intercropped systems and monoculture cultivars of *Panicum maximum* and Pigeon pea showed increases of 44.3% and 42.3% in N and P concentrations, respectively, compared to maize monoculture (**Figures 5A, C**). For the concentration of K (**Figure 5E**) and S (**Figure 5F**), it was observed that cultivars of *Panicum maximum* and Pigeon pea in monocultures and intercropped systems had similar concentrations, with average increases of 54.4% and 38.8% in K and S concentrations, respectively, compared to maize monoculture, which had the lowest concentrations (**Figures 5E, F**).

**Figure 6:**
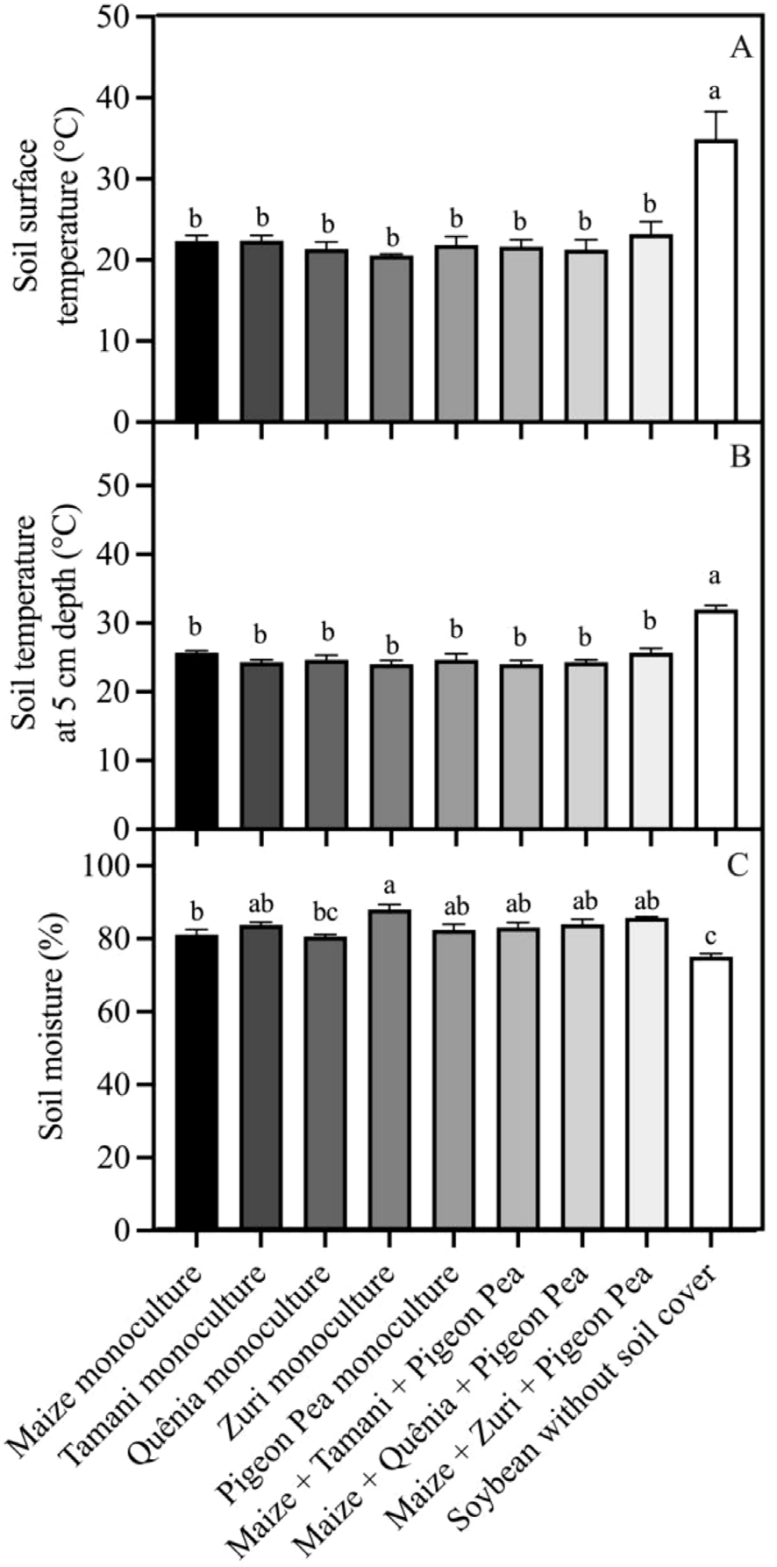
Soil microclimate conditions: Soil surface temperature **(A)**, soil temperature at 5 cm depth **(B)** and soil moisture **(C)**, registered in each integration strategy. Different lowercase letters between bars indicate statistical difference (p<0.05) between treatments by ANOVA followed by the Tuckey test. Stacked bars above the columns indicated the standard error of the mean. Each bar is the average value of 3 replicates (n =3).

**Figure 7:**
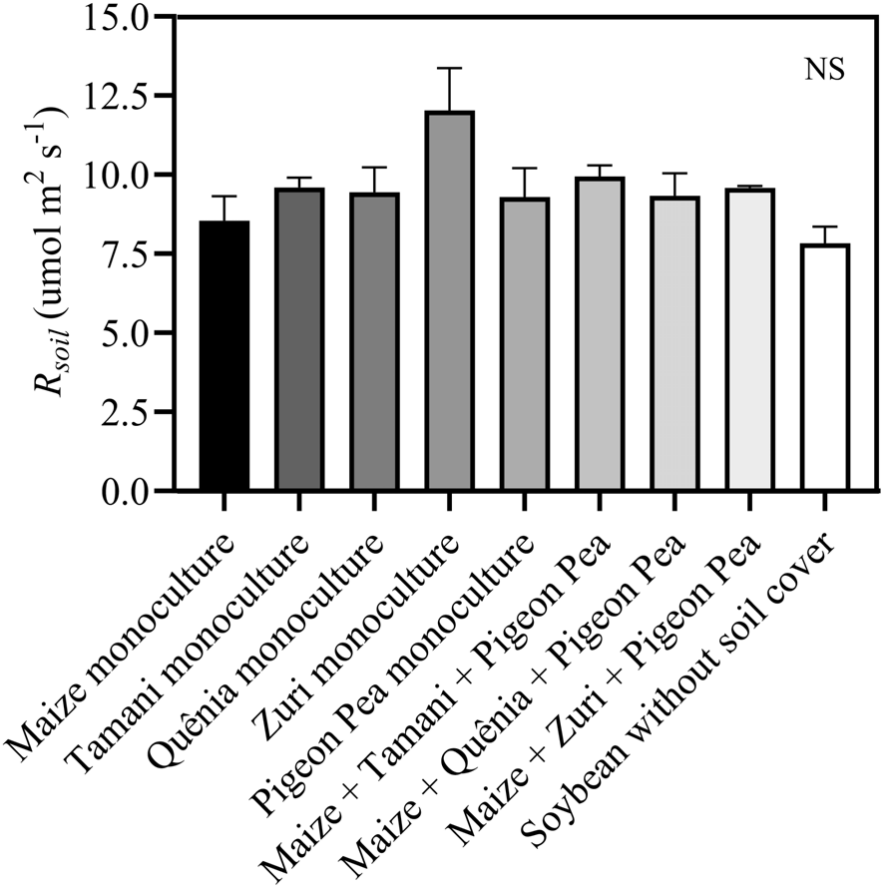
Soil respiration (*R_soil_*) measured in plots with soybean plants developed with or without crop residues produced by different integration strategies. NS in the top right corner indicates that there was no significant effect between the treatments by ANOVA followed by the Tuckey test. Stacked bars above the columns indicated the standard error of the mean. Each bar is the average value of 3 replicates (n =3).

Were observed that the soil surface temperature was, on average, 10°C higher (p<0.05) in plots without soil cover residues compared to those with crop residues covering the soil (**Figure 6A**). However, no differences were observed in the soil surface temperature among different integration strategies (**Figure 6A**). This same pattern was observed in the soil temperature measured at a depth of 5 cm (**Figure 6B**). Soil moisture was lower (p<0.05) in plots where soybeans were cultivated without soil cover compared to other treatments, except for Quênia guinea grass in monoculture (**Figure 6C**). Soil respiration (*R_soil_*) was not changed by treatments (**Figure 7**).

**Figure 8:**
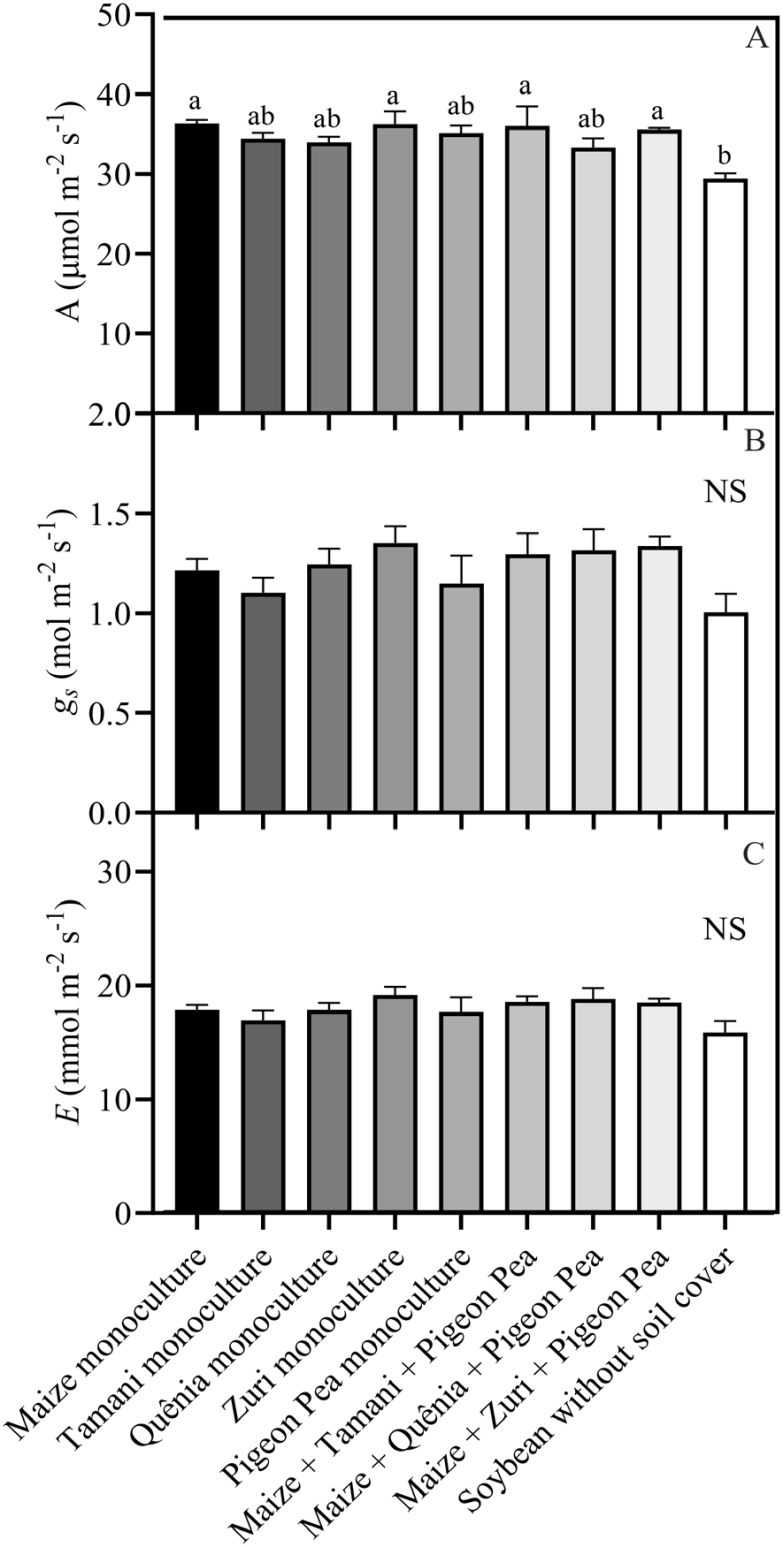
Leaf gas exchange parameters: *A* = net photosynthesis rate **(A)**, *g_s_* = stomatal conductance **(B)** and *E* = transpiration rate **(C)**, obtained from fully expanded leaves of soybean plants developed with or without crop residues produced by different integration strategies. Different lowercase letters between bars indicate statistical difference (p<0.05) between treatments by ANOVA followed by the Tuckey test; NS in the top right corner indicates that there was no significant effect between the treatments by ANOVA followed by the Tuckey test. Stacked bars above the columns indicated the standard error of the mean. Each bar is the average value of 3 replicates (n =3).

Leaf gas exchange measurements revealed that net photosynthesis rate (*A*) was influenced by the treatments (**Figure 8A**). Net photosynthesis rate was lower (p<0.05) in soybean plants cultivated without soil cover compared to maize monoculture, Zuri guinea grass monoculture, Maize + Tamani guinea grass guinea grass + Pigeon pea, and Maize + Zuri guinea grass guinea grass + Pigeon pea (**Figure 8A**). However, stomatal conductance (*g_s_*) and transpiration rate (*E*) were not affected by the treatments (**Figures 8B, C**).

**Figure 9:**
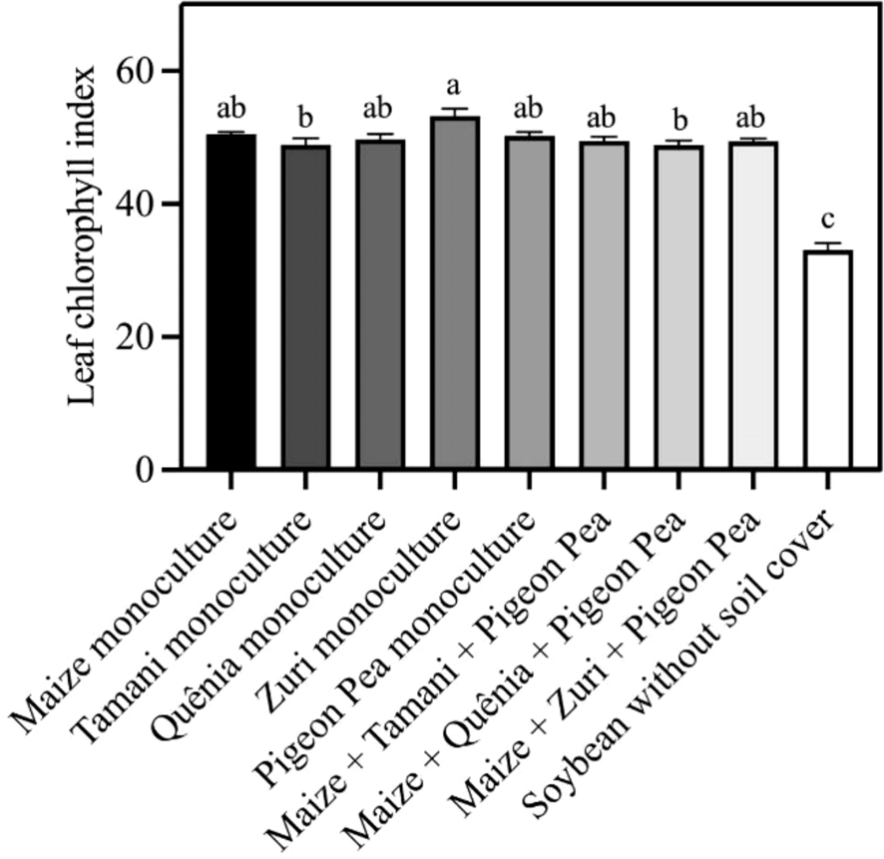
Leaf chlorophyll index obtained from fully expanded leaves of soybean plants developed with or without crop residues produced by different integration strategies. Different lowercase letters between bars indicate statistical difference (p<0.05) between treatments by ANOVA followed by the Tuckey test. Stacked bars above the columns indicated the standard error of the mean. Each bar is the average value of 3 replicates (n =3).

The highest concentration of leaf chlorophyll in soybean plants was observed in the treatment containing Zuri guinea grass residue in monoculture, while the lowest chlorophyll index was observed in soybean plants cultivated without soil cover (**Figure 9**). All integration strategies showed higher values of leaf chlorophyll when compared to soybean plants cultivated without soil cover.

**Figure 10:**
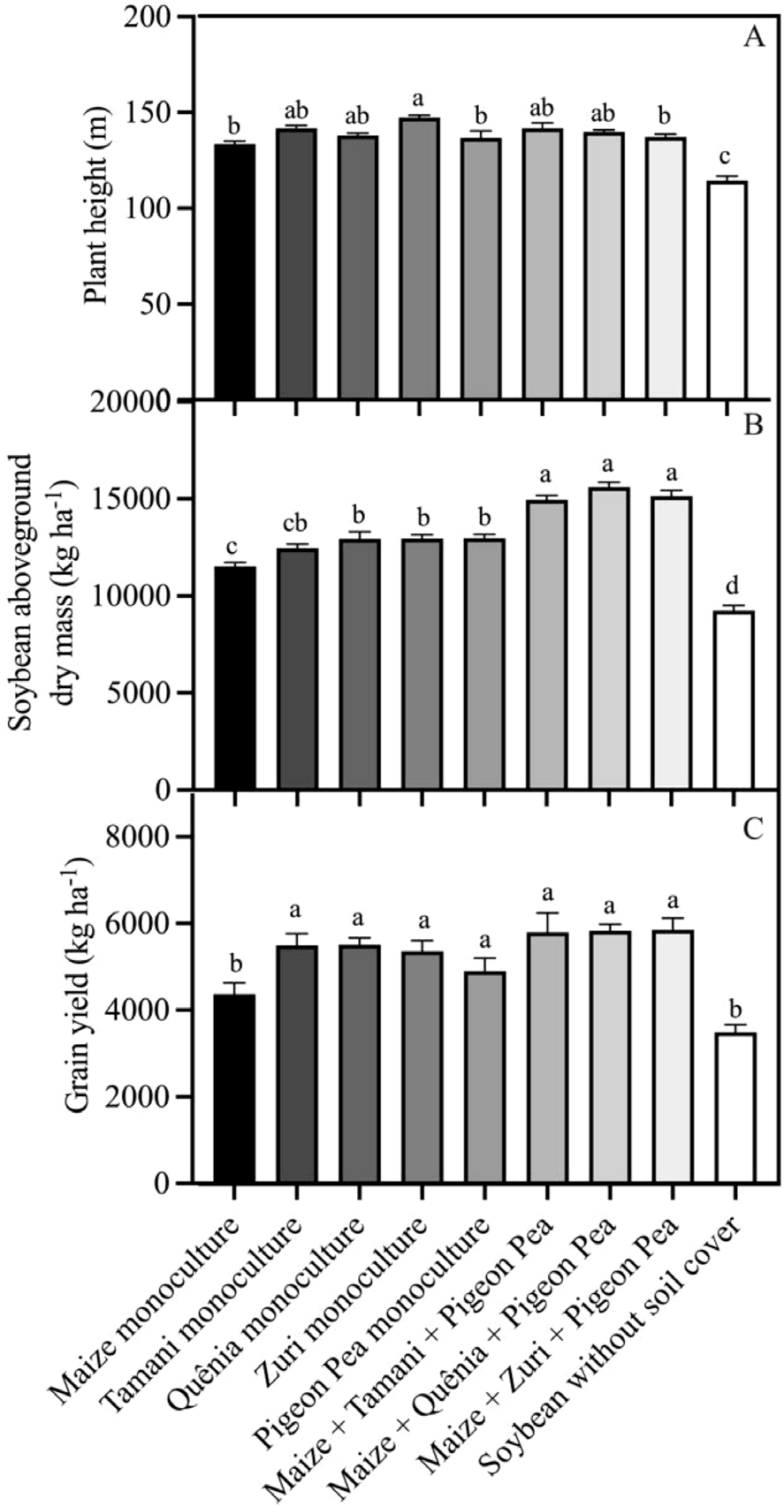
Plant height **(A)**, soybean aboveground dry mass **(B)**, and grain yield **(C)** obtained from soybean plants developed with or without crop residues produced by different integration strategies. Different lowercase letters between bars indicate statistical difference (p<0.05) between treatments by ANOVA followed by the Tuckey test. Stacked bars above the columns indicated the standard error of the mean. Each bar is the average value of 3 replicates (n =3).

Our integration strategies influenced plant height (**Figure 10A**). Soybean plants grown without soil cover exhibited the lowest height, while the tallest soybean plants were observed in the treatments: Tamani guinea grass guinea grass monoculture, Zuri guinea grass guinea grass monoculture, Maize + Tamani guinea grass guinea grass + Pigeon pea, and Maize + Quênia guinea grass + Pigeon pea (**Figure 10A**). The highest aboveground biomass productions of soybean plants were observed in the triple intercropping treatments, followed by Tamani guinea grass, Quênia, Zuri guinea grass guinea grass, and Pigeon pea in monoculture, which exhibited similar biomass levels. The lowest aboveground biomass production of soybeans was observed in plants cultivated without soil cover (**Figure 10B**). Cropping systems also influenced grain yield (**Figure 10C**). Systems including tropical forages (monoculture or triple consortium) showed increases in grain yield of 21.0% and 36.8% compared to monoculture maize and the conventional system (without soil cover biomass), which presented the lowest grain yield.

### 3.1 Multivariate analyzes

**Figure 11:**
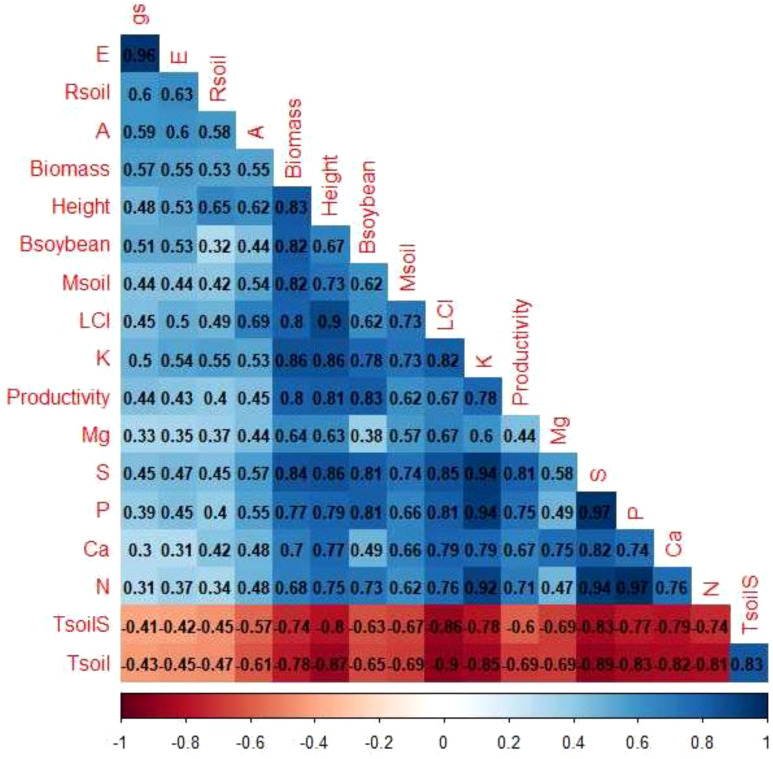
Pearson correlation (r) between parameters. Positive correlations are represented by blue backgrounds and negative correlations are represented by red backgrounds. Parameters: gs: stomatal conductance, E: transpiration rate, Rsoil: soil respiration, A: net photosynthesis rate, Biomass: soil cover biomass, Height: height of soybean plants, Bsoybean: aerial biomass of soybean, Msoil: soil moisture, LCI: leaf chlorophyll index, K: potassium concentration, Mg: magnesium concentration, S: sulfur concentration, P: phosphorus concentration, Ca: calcium concentration, N: nitrogen concentration, Tsoil: soil temperature (5 cm depth), TsoilS: temperature at the soil surface, Productivity: grain yield.

Through correlation analysis (**Figure 11**), the formation of two groups of variables was verified. Group 1 was composed of: Tsoil (soil temperature) and TsoilS (temperature at the soil surface) and Group 2 was composed of the other variables. The variables within each group showed positive correlations with each other and a negative correlation with the variables in the other group.

**Figure 12:**
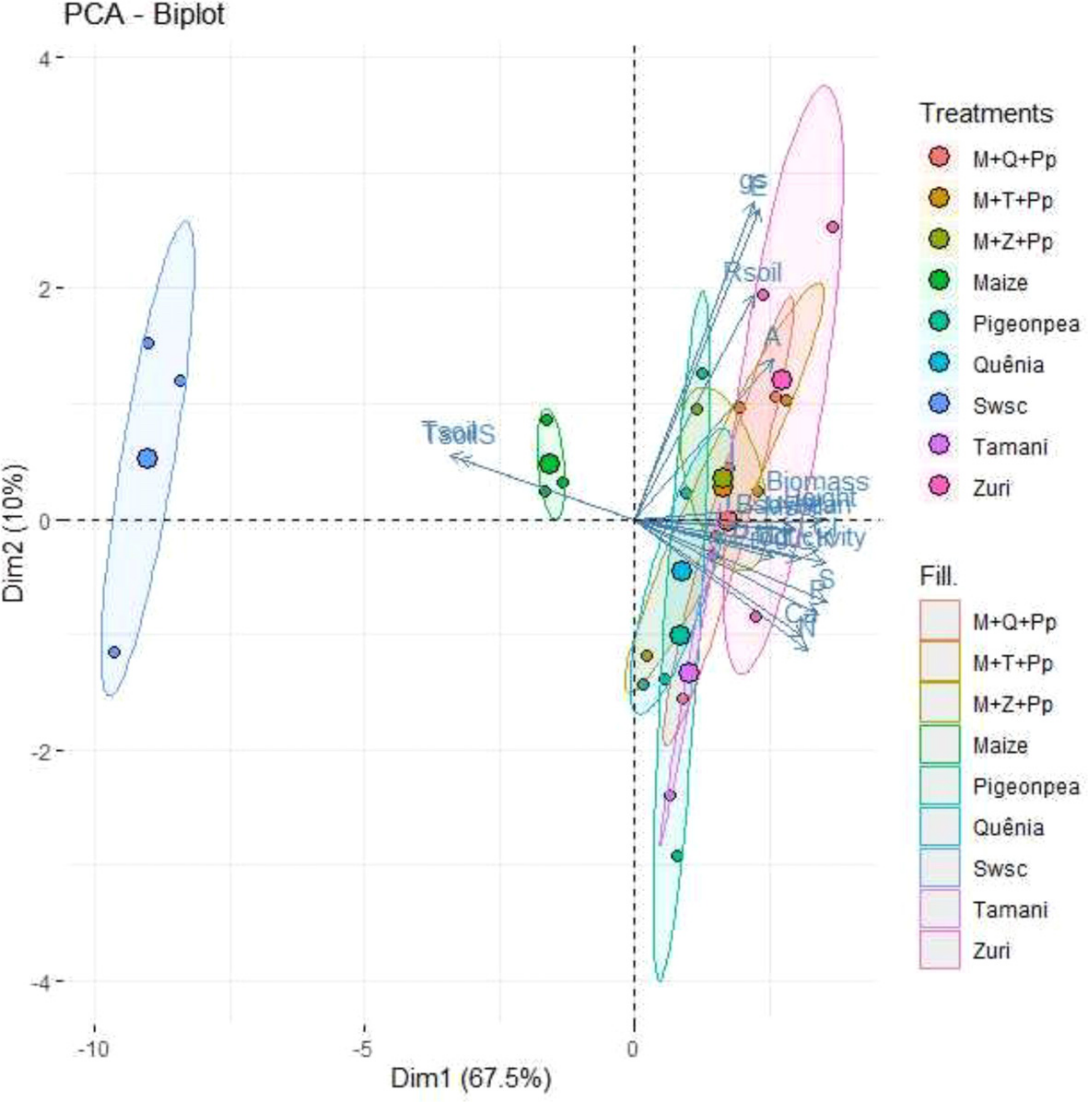
Two-dimensional PCA dispersion of 18 parameter scores, observations, and average values for biomass production, initial nutrient concentration in biomass, microclimate and soil respiration, physiological and soybean growth parameters. Treatments: maize (maize monoculture monoculture); Zuri guinea grass guinea grass (Zuri guinea grass guinea grass monoculture); Tamani guinea grass guinea grass (Tamani guinea grass guinea grass monoculture); Quênia guinea grass (Quênia guinea grass monoculture); Pigeon pea (Pigeon pea monoculture); M+Z+Pp (maize intercropped with Zuri guinea grass guinea grass and Pigeon pea); M+T+Pp (Tamani guinea grass guinea grass intercropped maize and Pigeon pea); M+Q+Pp (maize intercropped with Quênia guinea grass and Pigeon pea); Swsc (Soybean without soil cover). Parameters: gs: stomatal conductance, E: transpiration rate, Rsoil: soil respiration, A: net photosynthesis rate, Biomass: ground cover biomass, Height: soybean plant height, Bsoybean: soybean aerial biomass, Msoil: soil moisture, LCI: leaf chlorophyll index, K: potassium concentration, Mg: magnesium concentration, S: sulfur concentration, P: phosphorus concentration, Ca: calcium concentration, N: nitrogen concentration, Tsoil: soil temperature (10 cm depth), TsoilS: temperature at the soil surface, Productivity: grain yield.

Using the principal component analysis (PCA), were graphically understand the interrelationships between the variables through the first and second principal components (**Figure 12**). Were found that the first and second principal components jointly explained 77.5% of the total variation in the data. The first component (according to the displacement observed on the horizontal axis) explained 67.5%, presenting high and negative correlations with Tsoils and Tsoil, and high and positive correlations with Msoil (soil moisture), LCI (leaf chlorophyll index), Height (soybean plant height), Bsoybean (soybean aboveground dry mass), Biomass (ground cover biomass), K, Ca, and S and Productivity (grain yield). Second component (according to the displacement observed on the vertical axis) explained 10% of the data variation, presenting a high and negative correlation with gs (stomatal conductance) and *E* (transpiration rate).

The graphical analysis of PCA facilitated a comprehensive and objective understanding of the results (**Figure 12**), allowing us to discriminate among 3 treatment groups: 1 (Swsc - soybean without soil cover), 2 (Maize), and 3 (Pigeon pea, Quênia, Tamani guinea grass, Zuri guinea grass guinea grass, M+Q+Pp (maize intercropped with Quênia guinea grass and Pigeon pea), M+T+Pp (maize intercropped with Tamani guinea grass and Pigeon pea), and M+Z+Pp (maize intercropped with Zuri guinea grass and Pigeon pea). This demonstrates that the use of tropical forage crops in monoculture and triple intercropping shows higher plant residue production for soil cover, contributing to soil temperature reduction and moisture retention. The increased biomass production from the forage crops also showed a higher accumulation of nutrients (N, P, K, Ca, Mg, and S), positively influencing physiological aspects (LCI, *A*, *E*, *g_s_*), soybean growth parameters (Height, Bsoybean) and grain yield (Productivity) compared to soybean cultivation without soil cover (Group 1).

## 4 Discussion

Study aimed to compare the conventional soybean cultivation method with alternative regimes wherein soybean is grown over plant residues from prior tropical forage cultivation, either combined or not with maize or Pigeon pea plants. main hypothesis was corroborated since, the benefits of integrated systems on the physiology of soybean plants were detected. Furthermore, higher biomass production and nutrient concentration was observed when soybean was cultivated over crop residues originating from triple consortium through integration, which combined maize, a C4 grass, and a legume. In the following paragraphs, we discuss the potential implications of our results for more sustainable soybean cultivation.

Were observed variations in the production of plant residues by the different integrated crop-livestock regimes (**Figure 4**). The higher biomass production of Zuri guinea grass in both monoculture and intercropping is presumably related to its morphological characteristics, such as tall stature, rapid growth, high stem production (support structures), high dry matter yield, and vigorous regrowth during the off-season, leading to high production of plant residues (Silva et al., 2023a). On the other hand, Quênia guinea grass and Tamani guinea grass are grasses with intermediate and low statures, respectively (Tesk et al., 2020), explaining the intermediate biomass production results for soil cover (**Figure 4**).

It is important to note that the dry mass production of plant residues from the triple intercropping of maize + Tamani guinea grass + Pigeon pea was 14.5% higher than the monoculture of Tamani guinea grass, and for the triple intercropping with Zuri guinea grass and Quênia guinea grass, the production was similar to monocultures (**Figure 4**). The use of tropical forages (either as monocultures or in consortium) as cover crops is beneficial because, even under conditions of low or absent rainfall during off-season period (**Figure 2**), these forages show regrowth and adequate residue production for the no-tillage of soybean (Silva et al., 2024b). However, the presence of legumes in the system brings additional benefits such as increased carbon stocks in the soil (Han et al., 2024), biological nitrogen fixation (Jensen et al., 2020), organic matter input (Silva et al., 2022; Yan et al., 2023), improving physiological indices (Nasar et al., 2020) and increasing crop yields (Pereira et al., 2023).

The lower production of plant residues produced by maize in monoculture (**Figure 4**) is explained by the low quantity of residue left after harvest and by the early decomposition process that these residues undergo, as they remained in the field since harvest for ensilage (in May). On the other hand, Pigeon pea, due to lower precipitation (**Figure 2**), exhibited less regrowth, emitting fewer leaves (Ceballos et al., 2018), resulting in lower production of plant residues (**Figure 4**). Another important factor observed was that plant residues from maize and Pigeon pea monoculture did not provide adequate soil coverage (Dias et al., 2020; Santos et al., 2020), compared to systems with the presence of *Panicum* genus. These results demonstrate how integrated systems have the potential to increase the production of soil cover biomass, contributing to nutrient cycling (Oliveira et al., 2019), showing that the interspecific interactions in the triple intercropping are driven by plant-plant complementarity and cooperation rather than competition (Justes et al., 2021). Furthermore, the inclusion of Pigeon pea in the system presumably resulted in greater nitrogen input into the soil-plant system due to biological fixation and a gradual release of nutrients due to residue mineralization (Laroca et al., 2018), contributing to increased productivity of subsequent crops (Santos et al., 2020).

*Panicum* cultivars have been showing positive results in intercropping systems (Leal et al., 2023; Lima et al., 2023; Silva et al., 2023b, 2024c), providing soil improvements through decomposition, mineralization, and nutrient recycling. Additionally, the presence of Pigeon pea in the system contributes to increased soil nitrogen stocks through biological nitrogen fixation (Silva et al., 2022). Therefore, triple intercropping in the same area can be an important tool to combine the advantages of individually cultivated species (Pereira Filho et al., 2019). The higher concentrations of N and P obtained in the Pigeon pea residues cultivated in monoculture (**Figure 5**) are presumably associated with symbiotic association with bacteria from the *Rhizobium* genus, which facilitate N acquisition through biological fixation (Silva et al., 2020). As for the concentration of P, Wang and Lambers (2020) reported that cover crops, such as legumes, can mobilize labile P from the soil through mechanisms associated with the root system, rhizosphere pH, release of organic acids, phosphatase activity, and incorporate it into their biomass.

The biomass residues resulting from maize in monoculture showed lower concentrations of N, P, K, and S, due to the lower biomass production and consequently nutrient cycling. These results are consistent with studies conducted by Dias et al. (2020); Muniz et al. (2021), and Silva et al. (2023b), which compared integrated systems with the soybean/maize succession system. Tropical forage crops in monoculture or intercropped are efficient in reducing K losses through leaching (Silva et al., 2023b; Volf et al., 2023), being one of the most extracted nutrients by forage crops. Through their deep root system, they absorb K from the deeper soil layers, accumulate it in their biomass, and upon desiccation, release it on the surface, benefiting the succeeding crop (Costa et al., 2021). It is important to highlight that in soybean cultivated on the residues of different cropping systems, potassium fertilization was not applied, aiming to take advantage of the cycling of this nutrient. Silva et al. (2023b) observed that tropical forages used as soil cover crops can supply the amount of K required by the soybean crop, eliminating the need for mineral potassium fertilization.

The availability of S is influenced by the organic matter content present in the soil (Kumar et al., 2022), and integrated systems like those investigated in our research can be considered a highly efficient way to increase soil organic matter (Silva et al., 2023b), mainly due to the presence of Pigeon pea, as legume residues contain significant amounts of nitrogen that enhance carbon stabilization in organic matter (Silva et al., 2022). Therefore, the nutrient concentration per unit area in the integrated systems in our study is a good indicator of the performance of tropical forage crops as cover crops and the ecosystem services provided, reducing nutrient losses through leaching and/or runoff, maintaining soil moisture, and increasing nutrient cycling for the succeeding crop (Couedel et al., 2023).

Results showed that irrespective of the integrated crop-livestock system used (whether soil crop residues originated from a monoculture or a combination of three species), both the soil surface temperature and the temperature at a depth of 5 cm were lower when compared to conventional soybean cultivation. In some cases, this temperature difference exceeded 10°C compared to the conventional method. This difference was also observed in soil moisture, which was higher in all integrated systems, irrespective of the integration strategy used. Demonstrating the potential of cover crops to contribute to more favorable conditions (lower temperature and higher soil moisture) for the development of soybean plants, even in situations where rainfall distribution is inadequate (**Figure 2**). These results are widely supported by other studies and are related to increased shading and soil protection due to the presence of vegetation cover (Akhtar et al., 2019; Farias et al., 2020; Wang et al., 2021; Silva et al., 2024a).

Contrary to our expectation, soil respiration was not increased under an integrated system, since soil respiration is positively correlated with soil moisture and soil organic matter content but is often higher in warmer soils (Oliveira et al., 2020). This multifactorial interaction between edaphoclimatic conditions resulted in a similar carbon flux rate from soil to the atmosphere between integrated systems and conventional soybean cultivation, suggesting no additional carbon flux from soil to the atmosphere under integrated systems. Additionally, the higher deposition of organic matter under an integrated crop-livestock system on the soil promotes greater soil roughness, facilitating water flow between soil particles and maintaining soil moisture for longer periods (Akhtar et al., 2019). Some studies suggest that increased moisture in integrated systems protects plants against drought episodes, as the soil remains moist for longer, allowing stomata to remain open for extended periods, leading to greater carbon fixation and nutrient absorption compared to plants in traditional cultivation systems (Silva et al., 2024a).

Higher photosynthetic rates may be achieved when stomatal conductance (a measure of stomata openness) is higher (Kusumi et al., 2012). In the present study, no significant increase in stomatal conductance or transpiration rate of plants grown in integrated systems was observed, do not corroborate our main hypothesis. Thus, the photosynthetic gains observed in **Figure 8** are not related to a higher stomatal opening but rather to other metabolic factors.

Although stomatal conductance and transpiration rate were not altered in the present study, a significant increase in net photosynthesis rate was observed in some specific integrated systems. This increase is presumably related to the higher availability of nutrients originating from the degradation of plant residues (**Figure 5**). When released, these nutrients are absorbed by plants and become part of the chemical composition of various plant molecules. For example, N is often positively correlated with photosynthesis rates due to its high concentration in the enzyme Rubisco, the most abundant enzyme in plants and responsible for carbon fixation in the Calvin-Benson cycle (Nasar et al., 2020; Liu et al., 2023). P, in turn, is associated with photosynthesis because it is involved in the transfer and storage of energy, the synthesis of nucleotide molecules, and the functioning of various intermediate molecules in pathways related to photosynthesis (Dang et al., 2023). S is part of the composition of proteins, vitamins, and important enzymatic cofactors in photosynthesis; for example, ferredoxin acts as an electron carrier in the light reactions of photosynthesis (Taiz et al., 2017). K participates in the enzymatic activation of photosynthesis enzymes, in addition to activating photosystem II and being important in the process of carbohydrate transport (Johnson et al., 2022). All these nutrients were released in large quantities by the integrated systems, cumulatively impacting photosynthetic rates positively.

The greater availability of nutrients allows plants to invest resources in a more efficient photosynthetic apparatus (Silva et al., 2023b). This hypothesis was corroborated by the data presented in **Figure 9**, in which the leaf chlorophyll index was significantly higher in plants that grew in integrated systems, regardless of the type (in monoculture or combination). This presumably led to greater energy absorption, associated with a more efficient photosystem, higher electron transport rate, and energy dissipation. Several studies have found a positive correlation between both photosynthesis rate and chlorophyll index and photosynthesis and the concentration of N, P, K, and S (Singh et al., 2019; Latifinia and Eisvand, 2022; Silva et al., 2024b). These correlations were also observed in multivariate analyses in **Figure 11**.

Both the benefits of soil improvement and the benefits brought about in plant physiology and biochemistry by integration systems resulted in taller and more productive plants (Laroca et al., 2018; Lima et al., 2023; Vendramini et al., 2023). Were observed that soybean plants under the integrated systems had a greater height compared to those that grew under conventional cultivation conditions. While height did not show any response pattern among different types of integration (composed of a monoculture or three species), productivity showed a clear pattern.

The production of aboveground biomass was higher in triple integration systems, reaching values of approximately 15,000 kg ha^-1^, followed by monoculture integration systems, except maize. The lowest aboveground biomass productions were observed in the conventional soybean treatment. A possible explanation for the greater production of aboveground biomass of soybean in the triple intercropping is that this system combined the multiple benefits of individually grown crops, promoting less variation in soil temperature and preserving humidity (Muniz et al., 2021), favoring the best use of nutrients accumulated in the plants residue biomass (Kebede, 2021), throughout the soybean development cycle (Silva et al., 2024c), positively influencing growth and development of soybean plants.

The forage and triple intercropping monoculture systems showed greater potential for increasing soybean productivity, with increases of 21.0% and 36.8% compared to soybean grown in maize biomass and soil without cover residue, respectively. These results are associated with the importance of cover crops for increasing grain productivity, indicating a clear advantage of triple integration systems over conventional systems (Pariz et al., 2020; Kumari et al., 2023; Pereira et al., 2023).

It is worth noting that the benefits of integration systems are not only associated with improvements in soil climatic parameters or soil nutrition. The chemical diversity of compounds derived from the degradation of organic matter is of utmost importance for soil health and its microbiota growth (Serafim et al., 2023).

Studies show that greater species diversity in the integration system leads to higher productivity, as corroborated in the present study (Laroca et al., 2018; Pereira et al., 2023; Silva et al., 2024c). Additionally, according to compound diversity, the soil C pool can be favored, as well as the enzymatic activity of many important enzymes. Han et al. (2024), evaluating the consortium of maize and legumes (forage peanuts), observed an increase in the content of organic C and N in the soil compared to maize grown in monoculture. Carvalho et al. (2024) reported that a long-term crop-livestock integration system favors an increase in C levels in the soil. This greater chemical and allelochemical diversity also causes better weed and disease control, in addition to improving soil structure and water retention (Silva et al., 2020; Linhares et al., 2020; Silva et al., 2021).

## 5 Conclusion

The data presented in this study demonstrated that integrated systems, with the presence of grasses and legumes, can improve soil microclimate conditions and nutrient availability, enhancing soybean physiology through increased net photosynthesis rate and leaf chlorophyll index, resulting in greater plant height, aboveground biomass production, and soybean yield.

The results indicate that cultivars of *Panicum maximum* (Zuri, Tamani, and Quenia guinea grasses) and pigeon pea can be recommended for intercropping with maize, contributing to the sustainability of agricultural production. Furthermore, the findings are based on short-term results, and further investigation is strongly recommended regarding integrated systems with triple intercropping of maize with grasses and legumes and their effects on soil microclimate, nutrient availability, physiology, and long-term soybean productivity.

## Data Availability

The original contributions presented in the study are included in the article/supplementary material. Further inquiries can be directed to the corresponding author.

## References

[B1] Akhtar K. Wang W. Khan A. Ren G. Afridi M. Z. Feng Y.. (2019). Wheat straw mulching offset soil moisture deficient for improving physiological and growth performance of summer sown soybean. Agric. Water Manag 211, 16–25. doi: 10.1016/j.agwat.2018.09.031

[B2] Barbosa J. Z. Poggere G. Corrêa R. S. Hungria M. Mendes I. C. (2023). Soil enzymatic activity in Brazilian biomes under native vegetation and contrasting cropping and management. Appl. Soil Ecol. 190, 105014. doi: 10.1016/j.apsoil.2023.105014

[B3] Bhatti U. A. Bhatti M. A. Tang H. Syam M. S. Awwad E. M. Sharaf M. G.. (2024). Global production patterns: Understanding the relationship between greenhouse gas emissions, agriculture greening, and climate variability. Environ. Res. 245, 118049. doi: 10.1016/j.envres.2023.118049 38169167

[B4] Brito L. C. R. Souza H. A. Araújo Neto R. B. Azevedo D. M. P. Sagrilo E. Vogado R. F.. (2023). Improved soil fertility, plant nutrition and grain yield of soybean and millet following maize intercropped with forage grasses and crotalaria in the Brazilian savanna. Crop Pasture Sci. 74, 438–448. doi: 10.1071/CP22251

[B5] Carvalho A. M. Ramos M. L. G. Santos D. C. R. Oliveira A. D. Mendes I. C. Silva S. B.. (2024). Understanding the relations between soil biochemical properties and N2O emissions in a long-term integrated crop–livestock system. Plants 13, 365. doi: 10.3390/plants13030365 38337898 PMC10857650

[B6] Ceballos G. A. Fabian A. J. Silva J. C. O. Torino A. B. Bernardes G. F. (2018). Production and speed of decomposition of species of soil coverage in direct sowing system. Rev. Ciênc Agrár Amazon J. Agric. Environ. Sci. 61. doi: 10.22491/rca.2018.2631

[B7] Coelho A. E. Sangoi L. Sapucay M. J. D. C. Bratti F. Debiasi H. Franchini J. C.. (2023). Maize-ruzigrass intercropping, nitrogen fertilization and plant density improve the performance of soybean grown in succession. Rev. Bras. Eng. Agric. Ambient 27, 764–771. doi: 10.1590/1807-1929/agriambi.v27n10p764-771

[B8] Costa N. R. Andreotti M. Crusciol C. A. C. Pariz C. M. Bossolani J. W. Castilhos A. M.. (2020). Can palisade and Guinea grass sowing time in intercropping systems affect soybean yield and soil chemical properties? Front. Sustain Food Syst. 4. doi: 10.3389/fsusfs.2020.00081

[B9] Costa N. R. Andreotti M. Crusciol C. A. C. Pariz C. M. Bossolani J. W. Pascoaloto I. M.. (2021). Soybean yield and nutrition after tropical forage grasses. Nutr. Cycl Agroecosyst 121, 31–49. doi: 10.1007/s10705-021-10157-2

[B10] Couedel A. Alletto L. Justes E. (2023). The acquisition of macro-and micronutrients is synergistic in species mixtures: example of mixed crucifer-legume cover crops. Front. Agron. 5. doi: 10.3389/fagro.2023.1223639

[B11] Damian J. M. Matos E. S. Pedreira B. C. Carvalho P. C. F. Premazzi L. M. Cerri C. E. P. (2023). Intensification and diversification of pasturelands in Brazil: Patterns and driving factors in the soil carbon stocks. Catena 220, 106750. doi: 10.1016/j.catena.2022.106750

[B12] Dang K. Gong X. Liang H. Guo S. Zhang S. Feng B. (2023). Phosphorous fertilization alleviates shading stress by regulating leaf photosynthesis and the antioxidant system in mung bean (*Vigna radiata* L.). Plant Physiol. Biochem. 196, 1111–1121. doi: 10.1016/j.plaphy.2023.02.043 36931210

[B13] Delandmeter M. de Faccio Carvalho P. C. Bremm C. Cargnelutti C. S. Bindelle J. Dumont B. (2024). Integrated crop and livestock systems increase both climate change adaptation and mitigation capacities. Sci. Total Environ. 912, 169061. doi: 10.1016/j.scitotenv.2023.169061 38061655

[B14] Dias M. B. C. Costa K. A. P. Severiano E. C. Bilego U. Furtini Neto A. E. Almeida D. P.. (2020). *Brachiaria* and *Panicum maximum* in an integrated crop-livestock system and a second-crop maize system in succession with soybean. J. Agric. Sci. 158, 206–217. doi: 10.1017/S0021859620000532

[B15] Farias G. D. Dubeux J. C. B. Savian J. V. Duarte L. P. Martins A. P. Tiecher T.. (2020). Integrated crop-livestock system with system fertilization approach improves food production and resource-use efficiency in agricultural lands. Agron. Sustain. Dev. 40, 1–9. doi: 10.1007/s13593-020-00643-2

[B16] Gonzalez-Meler M. A. Silva L. B. C. Oliveira E. D. Flower C. E. Martinez C. A. (2017). Experimental air warming of a Stylosanthes capitata, vogel dominated tropical pasture affects soil respiration and nitrogen dynamics. Front. Plant Sci. 8, 46. doi: 10.3389/fpls.2017.00046 28203240 PMC5285360

[B17] Guo H. Xia Y. Jin J. Pan C. (2022). The impact of climate change on the efficiency of agricultural production in the world’s main agricultural regions. Environ. Impact Assess. Rev. 97, 106891. doi: 10.1016/j.eiar.2022.106891

[B18] Gut G. A. P. Emerenciano Neto J. V. Santo R. D. S. de Melo R. F. Nogueira D. M. Difante G. D. S.. (2022). Intercrops of grass with legumes as green manure for agroecological systems. Aust. J. Crop Sci. 16, 922–927. doi: 10.21475/ajcs.22.16.07.p3597

[B19] Han F. Javed T. Hussain S. Guo S. Guo R. Yang L.. (2024). Maize/peanut rotation intercropping improves ecosystem carbon budget and economic benefits in the dry farming regions of China. J. Environ. Manag 353, 120090. doi: 10.1016/j.jenvman.2024.120090 38301480

[B20] IPCC (2019). Climate change and land: an IPCC special report on climate change, desertification, land degradation, sustainable land management, food security, and greenhouse gas fluxes in terrestrial ecosystems. Eds. Shukla P. R. Skea J. Buendia E.C. Masson-Delmotte V. Pörtner H. O. Roberts D. C. Zhai P. Slade R. Connors S. Diemen R.v. Ferrat M. Haughey E. Luz S. Neogi S. Pathak M. Petzold J. Portugal Pereira J. Vyas P. Huntley E. Kissick K. Belkacemi M. Malley J.. Cambridge, United Kingdom and New York: Cambridge University Press.

[B21] Jensen E. S. Carlsson G. Hauggaard-Nielsen H. (2020). Intercropping of grain legumes and cereals improves the use of soil N resources and reduces the requirement for synthetic fertilizer N: A global-scale analysis. Agron. Sustain Dev. 40, 5. doi: 10.1007/s13593-020-0607-x

[B22] Johnson R. Vishwakarma K. Hossen M. S. Kumar V. Shackira A. M. Puthur J. T.. (2022). Potassium in plants: Growth regulation, signaling, and environmental stress tolerance. Plant Physiol. Biochem. 172, 56–69. doi: 10.1016/j.plaphy.2022.01.001 35032888

[B23] Justes E. Bedoussac L. Dordas C. Frak E. Louarn G. Boudsocq S.. (2021). The 4C approach as a way to understand species interactions determining intercropping productivity. Front. Agric. Sci. Eng. 8, 3. doi: 10.15302/J-FASE-2021414

[B24] Kebede E. (2021). Contribution, utilization, and improvement of legumes-driven biological nitrogen fixation in agricultural systems. Front. Sustain Food Syst. 5. doi: 10.3389/fsufs.2021.767998

[B25] Kumar U. Cheng M. Islam M. J. Maniruzzaman M. Nasreen S. S. Haque M. E.. (2022). Long-term Conservation Agriculture increases sulfur pools in soils together with increased soil organic carbon compared to conventional practices. Soil Tillage Res. 223, 105474. doi: 10.1016/j.still.2022.105474

[B26] Kumari V. V. Balloli S. S. Ramana D. B. V. Kumar M. Maruthi V. Prabhakar M.. (2023). Crop and livestock productivity, soil health improvement and insect dynamics: Impact of different fodder-based cropping systems in a rainfed region of India. Agric. Syst. 208, 103646. doi: 10.1016/j.agsy.2023.103646

[B27] Kusumi K. Hirotsuka S. Kumamaru T. Iba K. (2012). Increased leaf photosynthesis caused by elevated stomatal conductance in a rice mutant deficient in SLAC1, a guard cell anion channel protein. J. Exp. Bot. 63, 5635–5644. doi: 10.1093/jxb/ers216 22915747 PMC3444276

[B28] Laroca J. V. S. Souza J. M. A. Pires G. C. Pires G. J. C. Pacheco L. P. Silva F. D.. (2018). Soil quality and soybean productivity in crop-livestock integrated system in no-tillage. Pesqui Agropecu Bras. 53, 1248–1258. doi: 10.1590/S0100-204X2018001100007

[B29] Latifinia E. Eisvand H. R. (2022). Soybean physiological properties and grain quality responses to nutrients and predicting nutrient deficiency using chlorophyll fluorescence. J. Soil Sci. Plant Nutr. 22, 1942–1954. doi: 10.1007/s42729-022-00785-0

[B30] Leal V. N. Santos D. C. Paim T. P. Santos L. P. Alves E. M. Claudio F. L.. (2023). Economic results of forage species choice in crop–livestock integrated systems. Agriculture 13, 637. doi: 10.3390/agriculture13030637

[B31] Lima J. D. P. Torino A. B. Silva L. M. Nascimento Júnior L. F. Brito M. F. Costa K. A. P.. (2023). Crop-livestock integration improves physical soil, agronomic and environmental aspects in soybean cultivation. Plants 12, 3746. doi: 10.3390/plants12213746 37960102 PMC10647894

[B32] Linhares A. J. S. Gonçalves W. G. Cabral S. M. Brito M. F. Brandstetter E. V. Silva J. F. G.. (2020). Soil compaction affects sunflower and Paiaguas palisadegrass forage productivity in the Brazilian savanna. Aust. J. Crop Sci. 14, 1131–1139. doi: 10.21475/ajcs.20.14.07.p2294

[B33] Liu S. Wang L. Khan I. Nadeem F. Rehman A. (2023). Evaluating the influence of straw mulching and intercropping on nitrogen uptake, crop growth, and yield performance in maize and soybean. Front. Plant Sci. 14. doi: 10.3389/fpls.2023.1280382 PMC10611467 37900744

[B34] Ma B. Karimi M. S. Mohammed K. S. Shahzadi I. Dai J. (2024). Nexus between climate change, agricultural output, fertilizer use, agriculture soil emissions: Novel implications in the context of environmental management. J. Clean Prod 450, 141801. doi: 10.1016/j.jclepro.2024.141801

[B35] Malavolta E. Vitti G. C. Oliveira S. A. (1997). Avaliação do estado nutricional de plantas: princípios e aplicações. 2. ed (Piracicaba: Potafos: Potafos), 319p.

[B36] Mirzavand J. Asadi-Rahmani H. Moradi-Talebbeigi R. (2022). Biological indicators of soil quality under conventional, reduced, and no-tillage systems. Arch. Agron. Soil Sci. 68, 311–324. doi: 10.1080/03650340.2020.1832656

[B37] Muniz M. P. Costa K. A. P. Severiano E. C. Bilego U. O. Almeida D. P. Furtini Neto A. E.. (2021). Soybean yield in integrated crop–livestock system in comparison to soybean–maize succession system. J. Agric. Sci. 159, 188–198. doi: 10.1017/S0021859621000393

[B38] Nasar J. Shao Z. Arshad A. Jones F. G. Liu S. Li C.. (2020). The effect of maize–alfalfa intercropping on the physiological characteristics, nitrogen uptake and yield of maize. Plant Biol. 22, 1140–1149. doi: 10.1111/plb.13157 32609937

[B39] Nunes P. A. A. Laca E. A. Carvalho P. C. F. Li M. Souza Filho W. Kunrath T. R.. (2021). Livestock integration into soybean systems improves long-term system stability and profits without compromising crop yields. Sci. Rep. 11, 1649. doi: 10.1038/s41598-021-81270-z 33462356 PMC7813827

[B40] Oliveira S. M. D. Almeida R. E. M. D. Pierozan C. Reis A. F. D. B. Souza L. F. N. Favarin J. L. (2019). Contribution of maize intercropped with Brachiaria species to nutrient cycling. Pesqui Agropecu Trop. 49, e55018. doi: 10.1590/1983-40632019v4955018

[B41] Oliveira E. A. D. Manchon F. T. Ricketts M. P. Bianconi M. Martinez C. A. Gonzalez-Meler M. A. (2020). Plant diurnal cycle drives the variation in soil respiration in a C4-dominated tropical managed grassland exposed to high CO2 and warming. Plant Soil 456, 391–404. doi: 10.1007/s11104-020-04718-7

[B42] Pariz C. M. Costa N. R. Costa C. Crusciol C. A. C. Castilhos A. M. Meirelles P. R. L.. (2020). An innovative corn to silage-grass-legume intercropping system with oversown black oat and soybean to silage in succession for the improvement of nutrient cycling. Front. Sustain Food Syst. 4. doi: 10.3389/fsufs.2020.544996

[B43] Pereira R. R. Garcia I. M. Modesto V. C. Sekiya B. M. S. Soares D. D. A. Andreotti M. (2023). Soybean performance in succession to the intercropping of maize with marandu grass and pigeonpea in an integrated agricultural production system. Rev. Ceres 70, 72–80. doi: 10.1590/0034-737X202370030008

[B44] Pereira Filho A. Salviano A. M. Yuri J. E. Giongo V. (2019). Nutrient cycling in multifunctional agroecosystems with the use of plant cocktail as cover crop and green manure in the semi-arid. Afr J. Agric. Res. 14, 241–251. doi: 10.5897/AJAR2018.13600

[B45] Santos C. B. Costa K. A. P. de Souza W. F. Silva V. C. E. Brandstetter E. V. Oliveira S. S.. (2023). Production, quality of paiaguas palisadegrass and cattle performance after sorghum intercropping in pasture recovery in an integrated crop-livestock system. Aust. J. Crop Sci. 17, 361–368. doi: 10.21475/ajcs.23.17.04.p3700

[B46] Santos V. R. Costa L. C. Rocha A. M. S. Santos C. G. Santos M. A. L. Rabêlo F. H. S.. (2020). Biomass accumulation, extraction and nutrient use efficiency by cover crops. Res. Soc. Dev. 9, e9969109433. doi: 10.33448/rsd-v9i10.9433

[B47] Santos H. G. Jacomine P. K. T. Anjos L. H. C. Oliveira V. A. Lumbrerasm J. F. Coelho M. R.. (2018). Sistema Brasileiro de Classificação de Solos. 5th ed (Brasília: Embrapa: Embrapa CNPS).

[B48] Santos S. F. C. B. Souza H. A. Araújo Neto R. B. Sagrilo E. Ferreira A. C. M. Carvalho S. P.. (2021). Soil microbiological attributes and soybean grain yield in succession to maize intercropped with forage in the Maranhão eastern Cerrado. Int. J. Plant Prod 15, 669–677. doi: 10.1007/s42106-021-00167-z

[B49] Serafim M. E. Mendes I. C. Wu J. Ono F. B. Zancanaro L. Valendorff J. D. P.. (2023). Soil physicochemical and biological properties in soybean areas under no-till Systems in the Brazilian Cerrado. Sci. Total Environ. 862, 160674. doi: 10.1016/j.scitotenv.2022.160674 36493825

[B50] Silva J. A. G. Costa K. A. P. Severiano E. C. Silva A. G. Vilela L. Leandro W. M.. (2024b). Efficiency of desiccation, decomposition and release of nutrients in the biomass of forage plants of the genus Brachiaria after intercropping with sorghum in integrated systems for soybean productivity. Commun. Soil Sci. Plant Anal. 55, 1–19. doi: 10.1080/00103624.2024.2323076

[B51] Silva J. A. G. Costa K. A. P. Silva L. M. Severiano E. C. Silva F. G. Habermann E.. (2023b). Integrated systems improve the sustainability of soybean cultivation in the tropical region. Front. Sustain Food Syst. 7. doi: 10.3389/fsufs.2023.1224530

[B52] Silva J. A. G. Habermann E. Costa K. A. P. Silva L. M. Severiano E. C. Costa A. C.. (2024a). Integration crop-livestock system increases the sustainability of soybean cultivation through improved soil health and plant physiology. Agric. Ecosyst. Environ. 359, 108770. doi: 10.1016/j.agee.2023.108770

[B53] Silva A. A. Lacerda J. J. J. Carvalho S. P. Ferreira R. S. Brito R. R. Vogado R. F.. (2024c). Chemical and biological attributes of soil and soybean (Glycine max) yield in integrated systems in the Cerrado of north-east Brazil. Soil Res. 62, SR23120. doi: 10.1071/SR23120

[B54] Silva L. S. Laroca J. V. S. Coelho A. P. Gonçalves E. C. Gomes R. P. Pacheco L. P.. (2022). Does grass-legume intercropping change soil quality and grain yield in integrated crop-livestock systems? Appl. Soil Ecol. 170, 104257. doi: 10.1016/j.apsoil.2021.104257

[B55] Silva J. F. G. Linhares A. J. S. Gonçalves W. G. Costa K. A. P. Tormena C. A. Silva B. M.. (2021). Are the yield of sunflower and Paiaguas palisadegrass biomass influenced by soil physical quality? Soil Tillage Res. 208, 104873. doi: 10.1016/j.still.2020.104873

[B56] Silva C. H. D. L. Mello C. E. Silva J. O. D. Jakelaitis A. Marques R. P. Sousa G. D. D.. (2023a). Use of glyphosate in the management of Panicum maximum cv. BRS Zuri Guinea grass intercropped with maize. Rev. Bras. Eng. Agric. Ambient 27, 795–802. doi: 10.1590/1807-1929/agriambi.v27n10p795-802

[B57] Silva E. C. Muraoka T. Bastos A. V. S. Franzini V. I. Silva A. Buzetti S.. (2020). Nitrogen recovery from fertilizers and cover crops by maize crop under no-tillage system. Aust. J. Crop Sci. 14, 766–774. doi: 10.21475/ajcs.20.14.05.p2127

[B58] Silveira M. L. Cruz P. J. R. Vendramini J. M. B. Boughton E. Bracho R. Cardoso A. S. (2024). Opportunities to increase soil carbon sequestration in grazing lands in the southeastern United States. Grassl Res. 3, 69–78. doi: 10.1002/glr2.12074

[B59] Singh S. K. Reddy V. R. Fleisher D. H. Timlin D. J. (2019). Interactive effects of temperature and phosphorus nutrition on soybean: leaf photosynthesis, chlorophyll fluorescence, and nutrient efficiency. Photosynthetica 57, 248–257. doi: 10.32615/ps.2019.036

[B60] Soil Survey Staff (2014). Keys to soil taxonomy. 12th ed (Lincoln: United States Department of Agriculture, Natural Resources Conservation Service), 360 p.

[B61] Souza V. S. Santos D. D. C. Ferreira J. G. Souza S. O. Gonçalo T. P. Sousa J. V. A.. (2023). Cover crop diversity for sustainable agriculture: Insights from the Cerrado biome. Soil Use Manag 40, e13014. doi: 10.1111/sum.13014

[B62] Taiz L. Zeiger E. Møller I. M. Murphy A. (2017). Fisiologia e desenvolvimento vegetal. 6th ed (Porto Alegre: Artmed Editora).

[B63] Tesk C. R. Cavalli J. Pina D. S. Pereira D. H. Pedreira C. G. Jank L.. (2020). Herbage responses of Tamani Guinea grass and Quênia Guinea grass Guineagrasses to grazing intensity. Agron. J. 112, 2081–2091. doi: 10.1002/agj2.20189

[B64] Vendramini J. M. Silveira M. L. Moriel P. (2023). Resilience of warm-season (C4) perennial grasses under challenging environmental and management conditions. Anim. Front. 13, 16–22. doi: 10.1002/glr2.12074 PMC10575312 37841762

[B65] Volf M. R. Crusciol C. A. Kovar J. L. Rosolem C. A. (2023). Unraveling the role of ruzigrass in soil K cycling in tropical cropping systems. Nutr. Cycl Agroecosyst 126, 181–194. doi: 10.1007/s10705-023-10283-z

[B66] Wang Y. Lambers H. (2020). Root-released organic anions in response to low phosphorus availability: recent progress, challenges and future perspectives. Plant Soil 447, 135–156. doi: 10.1007/s11104-019-03972-8

[B67] Wang J. Zhang S. Sainju U. M. Ghimire R. Zhao F. (2021). A meta-analysis on cover crop impact on soil water storage, succeeding crop yield, and water-use efficiency. Agric. Water Manag 256, 107085. doi: 10.1016/j.agwat.2021.107085

[B68] Witcombe A. M. Tiemann L. K. Chikowo R. Snapp S. S. (2023). Diversifying with grain legumes amplifies carbon in management-sensitive soil organic carbon pools on smallholder farms. Agric. Ecosyst. Environ. 356, 108611. doi: 10.1016/j.agee.2023.108611

[B69] Yan Z. Zhou J. Liu C. Jia R. Mganga K. Z. Yang L.. (2023). Legume-based crop diversification reinforces soil health and carbon storage driven by microbial biomass and aggregates. Soil Tillage Res. 234, 105848. doi: 10.1016/j.still.2023.105848

